# Simultaneous Physico-Mechanical and In Vivo Assessment towards Factual Skin Performance Profile of Topical Polymeric Film-Forming Systems

**DOI:** 10.3390/pharmaceutics14020223

**Published:** 2022-01-18

**Authors:** Mirjana D. Timotijević, Tanja Ilić, Snežana Savić, Ivana Pantelić

**Affiliations:** Department of Pharmaceutical Technology and Cosmetology, University of Belgrade-Faculty of Pharmacy, Vojvode Stepe 450, 11221 Belgrade, Serbia; timotijevic.mirjana@gmail.com (M.D.T.); tanja.ilic@pharmacy.bg.ac.rs (T.I.); snezana.savic@pharmacy.bg.ac.rs (S.S.)

**Keywords:** formulation metamorphosis, skin irritation, friction, substantivity, hydroxypropyl cellulose, hydrophobic polymethacrylate copolymers, polyvinyl alcohol

## Abstract

Topical film-forming systems (FFS) change drastically after solvent displacement, therefore indicating their skin metamorphosis/transformation as a property of special regulatory and research interest. This paper deals with the lack of suitable characterization techniques, suggesting a set of methods able to provide a comprehensive notion of FFS skin performance. After screening the physico-chemical, mechanical and sensory properties of FFS and resulting films, an elaborate three-phase in vivo study was performed, covering skin irritation, friction and substantivity. Upon removal of 24-hour occlusion, no significant change in erythema index was observed, while the film-former type (cellulose ether, acrylate and/or vinyl polymer) affected transepidermal water loss (TEWL); hydrophobic methacrylate copolymer-based samples decreased TEWL by 40–50%, suggesting a semi-occlusive effect. Although both the tribological parameters related to the friction coefficient and the friction curve’s plateau provided valuable data, their analysis indicated the importance of the moment the plateau is reached as the onset of the secondary formulation, while the tertiary state is still best described by the completion of the film’s drying time. The final part of the in vivo study proved the high in-use substantivity of all samples but confirmed the optimal 4:1 ratio of hydrophobic cationic and hydrophilic polymers, as indicated during early physico-mechanical screening.

## 1. Introduction

In situ film-forming systems (FFS) represent an area of recent interest for (trans)dermal delivery, offering many advantages in terms of targeted treatment, with minimal lateral diffusion of the formulation and prolonged skin contact leading to less frequent application, ultimately improving the generally low adherence to topical treatments [1,2]. These thin polymeric films are nearly imperceptible to the patient’s eye, which, along with ease of application, entails additional practical advantages over conventional topical dosage forms [3,4].

Polymer selection appears to be determining the design of FFS, as it may advance the formulation’s structure and skin performance [5]. Despite the fact that some of the polymers used for the design of FFS are well-known excipients in dosage forms for other routes of administration (e.g., Eudragits gradually widened their application from oral solid dosage forms to topical delivery systems [6]), the evolution of formulation strategies has created a need for a range of new information.

For example, Gennari et al. studied Eudragit^®^ RL (ammonium methacrylate copolymer, 10%, 20% or 30%, *w/w*)-based FFS and the effect of solvents including mixtures of isopropyl alcohol and acetone in different ratios (90:10, 80:20, 70:30, 60:40 %, *v/v*), with or without a selected plasticizer on technological and biopharmaceutical properties [6]. Lunter et al. reported the successful development of film-forming emulsions and investigated the substantivity of these formulations [7,8,9]. Other authors tested different polymers (e.g., acrylates, acrylate/octylacrylamide copolymer, polyurethane-acrylates, cellulose derivatives, poly (vinyl pyrrolidones) and silicones) in FFS [1,3]. Among them, the use of methyl methacrylate copolymers appears of particular interest [1,10,11,12,13]. 

However, in many of these publications, the absence of data relating to certain properties gives rise to some uncertainty about the true characteristics of these matrices [12]. In the light of the recent regulatory guidelines on the quality and equivalence of topical products [14], quality target product profiles of formulations such as FFS will need to be supplemented with data obtained through specific characterization techniques [15].

Skin performance of topical formulations is an umbrella term encompassing not only certain safety and efficacy aspects, but also cosmetic criteria. In the case of FFS, the formulation’s metamorphosis or transformation upon application is the property of special interest, considering the fact that the formulation’s characteristics change drastically after solvent displacement, enabling film generation. The importance of formulation metamorphosis was recently rediscovered by both regulatory [14] and research communities [16,17,18,19]. Apart from the obvious impact on dermal bioavailability, the transformation of the drug delivery system during and after application can markedly affect its substantivity and tactile properties [19]. Depending on the excipient evaporation kinetics, the initial primary formulation morphs into the so-called secondary one, and further to a tertiary state, residing on the skin [17].

The diversity of indications for FFS topical treatments implies the need for comprehensive research, going way beyond the physicochemical requirements to include the in vivo assessment of the biophysical properties of the skin [19,20,21]. However, as the existing standard methods, i.e., ISO and ASTM guidelines [22,23,24,25], are mainly designed for testing thin films produced by and used in industries other than the pharmaceutical industry, this paper deals with the obvious lack of characterization techniques suitable for topical films, suggesting a set of methods aiming to provide a comprehensive notion of FFS skin performance.

Taking into consideration the number and versatility of polymers available for pharmaceutical development [26], this paper deals with common representatives from the group of cellulose ethers, acrylates and vinyl polymers, all acknowledged for their film-forming capacity. Apart from the variation in the film-forming polymer or their combinations, the design space included a selection of plasticizers (triethyl citrate (TEC), tributyl citrate (TBC), propylene glycol or glycerol, with optional use of potential penetration enhancers) and suitable solvents (ethanol 96%, propylene glycol, isopropyl alcohol, ethyl acetate or combinations thereof). The first phase of the study involved a set of optimized physico-mechanical approaches that discerned the formulations with desired drying time, film thickness and low stickiness. This significantly refined the number of samples entering the in vivo skin performance studies. To the best of our knowledge, this work is a unique contribution to the field of topical FFS, simultaneously considering in vivo skin biophysical parameters, tribological properties (friction) and in-use substantivity as a set of techniques, leading to a better understanding of their complex skin performance.

## 2. Materials and Methods

### 2.1. Materials

Hydrophilic polymer, Klucel^®^ GF (hydroxypropyl cellulose, HPC), was supplied by Caesar & Loretz GmbH (Hilden, Germany). Hydrophobic polymethacrylate copolymers, Eudragit^®^ NE 30 D (poly (ethyl acrylate-co-methyl methacrylate) 2:1 (polyacrylate dispersion 30% Ph. Eur.)) and Eudragit^®^ RS PO (poly (ethyl acrylate-co-methyl methacrylate-co-trimethylammonioethyl methacrylate chloride), i.e., copolymer type B, Ph. Eur.), were obtained from Evonik Rohm GmbH (Darmstadt, Germany). Serving as the polymer in the control formulation for the in vivo part of the study, polyvinyl alcohol (PVA) was purchased from Sigma Aldrich (Schnelldorf, Germany). TEC and TBC were supplied by Merck GmbH (Darmstadt, Germany). As potential penetration enhancers, medium-chain triglycerides (Miglyol^®^ 812, MCT, purchased from Fagron, Trikala, Greece) and polysorbate 80 (from Sigma Aldrich, Schnelldorf, Germany) were varied. Glycerol and propylene glycol were supplied by Carl Roth GmbH (Karlsruhe, Germany). All the solvents used were of pharmaceutical grade.

### 2.2. Preparation of Polymeric FFS

The samples were prepared as follows: the chosen film-forming polymer was dissolved in the selected solvent or a mixture of solvents, as specified in Table 1, and the solution was mixed (magnetic stirrer RCT basic, IKA^®^, Staufen, Germany) until complete dispersion of the polymer occurred (typically 2.5–3 h, apart from the HPC, which required continuous stirring for up to 24 h). The following day, the selected plasticizer was added, and the mixing was continued at 900 rpm until complete homogenization. All formulations were stored in tightly closed dark glass bottles.

### 2.3. Screening of Physico-Chemical, Mechanical and Sensory Properties

During the first phase of the study, the formulations were subjected to a series of specifically optimized characterization methods in order to obtain insight into the following properties: drying time, thickness, flexibility and sensory properties of the casted films, as well as spreadability and pH of the film-forming systems.

#### 2.3.1. Drying Time

A pre-defined quantity of each sample (500 μL) was uniformly distributed in Petri dishes (18 cm^2^) using a micropipette. The drying time of the films was measured with a stopwatch in triplicate, both at room temperature and at 32 °C by touching the film surface. The first part of the test was conducted with careful control of the ambient conditions (temperature 23 ± 2 °C, relative humidity 40 ± 10%). Secondly, in order to relate to in-use drying of the samples (average skin temperature), the test was performed in the Orbital Shaker Incubator ES 20 (Biosan, Riga, Latvia). The temperature of the device was set to 32.0 ± 0.1 °C well in advance of the experiment. If any remaining liquid was still apparent upon tactile assessment, the experiment was repeated with prolonged drying time. The obtained mean drying times were expressed in min ± standard deviation (SD) and graded as low (≤5 min), medium (5–7 min) or high (>7 min), according to [3].

#### 2.3.2. Sensory Properties

This part of FFS profiling was abridged to include the visual appearance and stickiness of the generated films. The visual appearance of the films formed both after drying at room temperature and at 32 °C was regarded with and without a magnifying glass, and descriptively estimated as “transparent” (i.e., almost invisible film); a film which is “not fully transparent” or “mildly turbid”; or a film which is “non-transparent” or “turbid”.

The stickiness of the film was evaluated in triplicate by applying a piece of cotton bud under low pressure on the dry film [3]. The fact that the cotton bud was weighed on an analytical balance (ABJ 120-4M, Kern & Sohn GmbH, Balingen, Germany) both before and after application allowed the stickiness to be rated as “low” (no observable cotton fibers on the film surface), “medium” (a thin layer of fiber filaments is visible, with 0.01 g as the cut-off value of the retained cotton) and “high” (a thicker layer of fiber filaments is observed, with 0.02 g as the cut-off value of the retained cotton).

#### 2.3.3. Film Thickness

The test was performed using a digital micrometer compliant with DIN 863, supporting the range of thickness measurements 0–25 mm/0.001 mm (Kern, Balingen, Germany), by applying 10 μL of the samples to the microscope glass plate as the substrate. Before applying a test sample, the thickness of each glass plate was measured at 3 different points. After complete sample drying, the thickness of the film formed on the glass plate was measured and corrected with the thickness of the clean substrate. Each sample was tested in triplicate, allowing the mean values to be expressed in mm (± standard deviation, SD).

#### 2.3.4. Spreadability

Testing was performed by applying 10 μL of each sample with a micropipette perpendicularly to the surface of a microscopic glass plate. After the film dried at 23 ± 2 °C, its diffusion area was determined using millimeter graph paper, i.e., by placing the paper under the glass plate and counting the number of occupied squares with the aid of a magnifying glass. The obtained values were expressed in mm^2^ ± SD as a mean value of three consecutive measurements.

#### 2.3.5. Flexibility/Mechanical Resistance

As the key parameter of a film’s mechanical resistance, flexibility was evaluated with the so-called folding technique. The folding endurance value could be defined as the number of times a film can be folded at the same place without breaking. This test denotes the brittleness of the film (the lower the folding endurance value, the more brittle the film is), allowing the assessment of a film’s integrity [27,28].

The testing protocol was adjusted to suit dermal FFS in the following way: 500 μL of each sample was applied using a micropipette, and uniformly distributed over a length of 4 cm onto a 27 × 4.4 cm rubber substrate. After the film dried, the rubber band was repeatedly rolled and unrolled over the whole length of the film. After each rolling cycle, the film was inspected for cracking or other specific changes using a magnifying glass. The resulting number of folds defined as the “folding endurance value” was expressed as the number of rolling times up to the first perceived, visible change in the film on the rubber substrate, which relates to the film’s proneness to cracking. The test was performed in triplicate, and average folding values ± SD were reported.

#### 2.3.6. pH Value of the FFS

The pH of a delivery system may affect both the state of a model drug and the skin’s buffering capacity, possibly leading to changes in subsequent skin partitioning [17]. Therefore, screening of FFS pH values was performed by direct immersion of the previously calibrated pH checker^®^ HI98103 probe (Hanna Instruments, Woonsocket, RI, USA). Testing was carried out in triplicate, resulting in mean values of three individual measurements (± SD).

### 2.4. In Vivo Skin Performance Studies

#### 2.4.1. Subjects

The study was performed after obtaining written informed consent from the volunteers and permission from the Ethical Committee of the Faculty of Pharmacy, University of Belgrade, Serbia (approval number: 2073/2). During the study, 16 healthy female volunteers (8 for skin irritation study and 8 for tribological study) aged 33 ± 8 years and without previous history or clinical signs of dermatological diseases, physical impairments of the skin or allergic reactions participated in the evaluation of the selected FFS. The volunteers were instructed not to use any skin or body care product on their arms 48 h before the beginning of the study and were not allowed to wash the treated areas 24 h before or throughout the study. Furthermore, the consumption of coffee or tea was denied at least 3 h before and throughout the study. Volunteers were acclimatized to the controlled room conditions (temperature 22 ± 1 °C and relative humidity 40 ± 5%) at least 30 min prior to the appropriate measurements.

#### 2.4.2. Study Protocols

**Figure 1:**
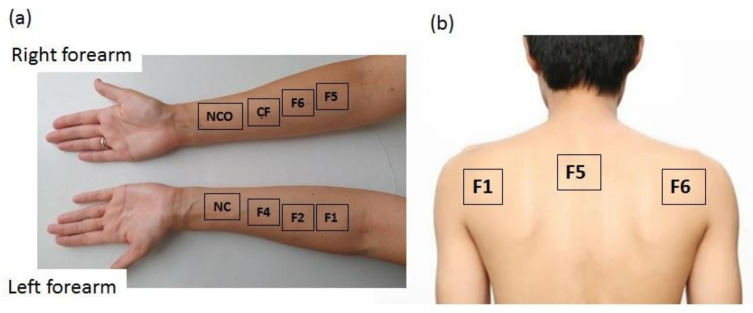
Actual layout of the samples and control sites: non-treated control under occlusion (NCO) and without occlusion (NC) within (**a**) in vivo study under occlusion and (**b**) in vivo substantivity study.

In the first part of the study, to estimate the possible skin irritation effects of the selected FFS, the following skin biophysical parameters were assessed: erythema index (EI) by Mexameter^®^ MX 18 (expressed in arbitrary Mexameter^®^ units, potentially ranging from 0 to 999 for erythema), transepidermal water loss (TEWL) using Tewameter^®^ TM 210 (units g/h/m^2^), stratum corneum hydration (SCH; arbitrary capacitance-based Corneometer^®^ units) and skin pH value by Cutometer^®^ MPA 580 (all from Courage+Khazaka Electronic GmbH, Köln, Germany). These parameters were measured prior to (baseline values) and 1 h after the 24 h occlusion, and residual films were removed. Specifically, a quantity of 5.5 μL/cm^2^ of each of the test samples was applied with a micropipette on the volar side of the volunteers’ forearms using a precisely marked cardboard ruler with three empty rectangular spaces (each 9 cm^2^, 3 cm × 3 cm) so that the polymeric system was applied perpendicularly to the skin surface. After complete drying of the applied samples, i.e., formation of in situ polymeric films on the skin, the pre-determined treated surfaces were immediately covered with silicone film (Parafilm^®^ M, Brand GmbH, Wertheim, Germany) and fixed with hypoallergenic adhesive plaster Vivafix^®^ (Tosama, Domžale, Slovenia). The test site adjacent to the left wrist represented the non-treated control (NC), while the site next to the right wrist was occluded as described above and served as the non-treated control under occlusion (NCO). The study design is depicted in Figure 1a.

In the second phase of the in vivo study, the skin friction coefficient (expressed as arbitrary torque-based Frictiometer^®^ units) was determined using a Frictiometer^®^ FR700 (Courage+Khazaka) equipped with a plain, smooth Teflon (PTFE) disk. Measurements of skin friction value belong to the sphere of tribology and were performed at 90 rpm for 100 s, with one measurement taken per second. Prior to the first skin contact, continuous measurements were performed in the air for at least 15 min in order to warm up the probe of the device and to obtain more reproducible results. A quantity of 5.5 µL/cm^2^ of each tested sample was applied with a micropipette on the volar side of forearms using a precisely marked cardboard ruler with three empty circular spaces (each being 12.56 cm^2^) so that the polymer solution was applied perpendicularly to the skin. A defined site on the left arm served as the non-treated control (NC). The measurements were conducted at the following time points: after 5 min, after 30 min, after 1 h, after 6 h and after 24 h of the application of the selected FFS without occlusion, to determine the friction coefficient in various stages of FFS transformation during their skin application, leading to the formation of the residual film on the skin, and its integrity. The device’s accompanying MPA-Software converted the rapidly obtained data into arbitrary units (A.U.).

The final part of the in vivo study was conducted according to a modification of Schmidberger et al. [29] and aimed to more specifically target film substantivity. For that purpose, the investigated FFS were loaded with 0.9% of avobenzone, a UV filter with negligible skin penetration potential [30]. In total, 5.5 µL/cm^2^ of FFS was applied to the marked 9 cm^2^ square sites on the backs and upper arms of the volunteers (Figure 1b). After complete drying of the films, a piece of cloth (6 × 6 cm) was fixed on the inner side of the volunteers’ clothing with safety pins, completely covering the generated film. After that, the volunteers were allowed to return to their normal activities for the next 3 h. Upon their return, the formulations were washed away using two consecutive cotton swabs each soaked with 2 mL of isopropyl alcohol. The two swabs were collected in a centrifuge tube and 10 mL of isopropyl alcohol was added to extract the avobenzone. The avobenzone potentially transferred to the cloth was extracted in a second centrifuge tube with 14 mL of isopropyl alcohol. Subsequently, the tubes were shaken for 1 min and then exposed to an ultrasonic bath for 50 min while maintaining the temperature below 20 °C in order to avoid the thermal decomposition of avobenzone. The amount of avobenzone was determined using a UV/Vis Spectrophotometer Evolution 300 (Thermo Scientific, Waltham, MA, USA) at 360 nm. Throughout the extraction procedure and subsequent analysis, the aliquots were protected from direct light. The recovery of avobenzone ranged from 72 to 123% of the applied amount.

### 2.5. Statistical Analysis

Where applicable, data were presented as mean ± SD. The results of the characterization of the tested FFS were statistically compared using Student’s *t*-test or one-way analysis of variance (ANOVA). The results of the in vivo skin performance study were either analyzed by Student’s *t*-test or ANOVA followed by the Tukey post hoc test or by the nonparametric Kruskal–Wallis test followed by the Wilcoxon Signed-Rank Test or Mann–Whitney U test for pairwise comparisons between groups. An assessment of the normality of data was carried out using the Shapiro–Wilk test. Statistical analyses were performed using PASW Statistics software package, version 18.0 (SPSS Inc., Chicago, IL, USA). The level of significance was set to *p* < 0.05.

## 3. Results and Discussion

### 3.1. Preformulation Study

Around 50 different formulations were prepared and submitted through a preformulation study (described within the Supplementary Materials) comprising a hydrophilic (HPC) and two hydrophobic (Eudragit^®^ NE 30 D (hereafter NE) and Eudragit^®^ RS PO (hereafter RS)) film-forming polymers. The hydrophobic polymers were selected to include a neutral (NE) and a cationic (RS) acrylate copolymer. Although all the FFS samples were screened for relevant physico-chemical and mechanical properties, the following parameters were accepted as the first cut-off for further assessment: drying time at room temperature < 60 min; drying time at skin temperature < 15 min; low stickiness of the formed films at 32 °C and at room temperature; film thickness < 0.1 mm; and pH in the range of 5.5–8.5. This allowed the number of prospective formulations to be reduced to 20. In the second tier of refinement, folding endurance value < 15 was set as the main exclusion criteria, which enabled a more rational selection of five promising formulations for subsequent in vivo skin performance testing. As presented in Table 2, the selected formulations comprised the polymers RS at 8.5%, 10% and 17.5%, NE at 6.0% and HPC at 3.5%, which is in line with the previous research in the field [1,3,21]. The fact that polymer RS dominated the chosen samples is related to its cationic nature and expectedly favorable skin substantivity.

As plasticizers suitable for RS dispersions (20% relative to the dry weight of the polymer, according to Garvie-Cook et al. [13]), propylene glycol and TEC were discerned, while the use of glycerol or MCT failed to provide films with sufficient flexibility. The use of isopropyl alcohol as a solvent for the polymers RS (10%) or NE (5%) did not provide satisfactory results in terms of film stickiness, irrespective of the drying conditions. When combined with NE (specifically 6% or 8.5%), the same solvent afforded low stickiness of the films at 32 °C and at room temperature, but in turn reduced their flexibility. As for the HPC-based formulations, the samples comprising 4%–5% *w/w* of the polymer were attributed with a marked increase in viscosity after 3.5 months of storage and were therefore unsuitable for further analysis.

### 3.2. Physico-Mechanical and Sensory Profiling

A comparative view of the obtained drying times (Table 3) favored the use of an incubator chamber, which significantly shortens the test while providing results more relevant to the in-use conditions. Among the tested FFS, the formulation F2 (stabilized with 10% w/w of polymer RS) was attributed with a significantly faster drying time when compared to formulations containing a lower amount of the same polymer (sample F1 with 8.5% RS), polymers NE or HPC. Interestingly, the tested polymer combination within sample F6 resulted in the same mean drying time as in the case of F2, implying that the addition of 1% of the hydrophilic polymer (in this case, HPC) allows for a considerable reduction in the share of the hydrophobic polymer (i.e., from 10% to 4% of RS). Thus, the selected formulations could be classified into three groups according to the drying time at skin temperature: the group with drying times ≤ 5 min (F2, F6); the group within the range 5–7 min (F1, F3, F5); and the group with drying times > 7 min (only F4).

It is interesting to note that the increase in the concentration of the polymer RS from 10% (F2) to 17.5% (F3) failed to linearly affect the drying time of the film at 32 °C. On the other hand, the addition of polysorbate 80 at 1% *w/w* (sample F1) obviously played a part in the protraction of the film drying time at 32 °C to 6 min 40 s. Therefore, the amount of this non-ionic surfactant should be lowered, considering the favorable drying time of the sample F6 containing 0.3% of polysorbate 80, which would still be sufficient for most of its intended functions (e.g., solubilization or penetration enhancement).

While increasing the concentration of HPC from 2.5% to 3.5% (resulting in sample F4), a significant increase in the film drying time at 32 °C occurred. This, along with the extensive stirring time of HPC dispersions, is another unfavorable property of this hydrophilic polymer, possibly related to its high molecular weight [31].

Film-forming formulations are among several dermal delivery systems that require simultaneous discussion of certain sensory and mechanical properties. With respect to aesthetic attributes, F1 and F3 appeared to be the formulations of choice because they formed transparent, homogeneous films with low stickiness after drying at both 32 °C and at room temperature. While samples F2, F5 and F6 were attributed with mild turbidity, only the sample F4 containing HPC as the principal film-former was rated with intensive turbidity (white color), possibly influencing prospective patient adherence. All the tested samples were attributed with low stickiness. As for the elementary FFS spreadability, the increase in the amount of the polymer RS expectedly led to a decrease in the resulting film surface (Table 3). Nevertheless, the lowest spreading was found for the sample based on HPC, despite the fact that it was attributed with one of the highest drying times in ambient conditions (40.0 ± 0.6 min).

The results of FFS characterization in terms of folding endurance value, film thickness and pH value are presented in Table 4. The formed films were attributed with overall satisfactory thickness, ranging from 0.007 to 0.096 mm, which is unlikely to compromise patient adherence. As expected, the increase in the amount of RS polymer resulted in a gradual increase in generated film thickness. However, the thickness itself cannot serve as a measure of the film’s mechanical properties as the sample F1 with the thinnest residual film was in fact attributed with the most favorable flexibility. The observations related to the samples F1–F3, based on the same film-former (8.5–17.5% *w/w* of hydrophobic Eudragit^®^ RS PO), illustrate the importance of FFS optimization for where desired film properties can be attained even with lower film-forming polymer concentrations. Additionally, the combination of this polymer with 1% *w/w* of the hydrophilic HPC proved to be a good option, with highly satisfactory resistance to mechanical stimuli. Such findings may ultimately lead to more cost-effective therapeutic approaches.

Formulation studies in the era of skin microbiome-oriented approaches [32] pay more attention to the pH values of both the skin and topical samples [33]. This is especially the case for prospective film-forming systems envisioned for prolonged skin contact. The pH values of the tested FFS were in the range 6.1–8.3 (Table 4). While the formulations F1, F2, F3 and F5 were attributed with nearly neutral pH values, generally acceptable for either healthy or diseased skin, the sample F4 demonstrated a somewhat higher pH of 8.3 ± 0.1. Although this basic pH may not be universally desired, according to the producer’s documentation, it is expected to maintain good formulation stability in storage [34].

The obtained results enabled a better understanding of FFS physico-mechanical properties and allowed for additional sample refinement prior to the in vivo phase of the study. The cost–benefit analysis indicated that sample F3, comprising the highest amount of the polymer RS (17.5% *w/w*), may be omitted from further experiments. Meanwhile, an additional control formulation (CF) based on PVA (10% aqueous dispersion with 0.5% of propylene glycol) as a reference hydrophilic film-former was included [35].

### 3.3. In Vivo Skin Performance

When developing novel topical formulations, the assessment of skin irritation potential is required as this is one of the most common side effects. In the first phase of the skin performance study, we evaluated the in vivo irritation potential of pre-selected placebo film-forming formulations (F1, F2, F4, F5, F6 and CF) by means of EI measurements after 24 h occlusion. Additionally, in order to gain further insight into skin performance, TEWL, SCH and skin pH were also measured as biophysical parameters of interest.

**Figure 2:**
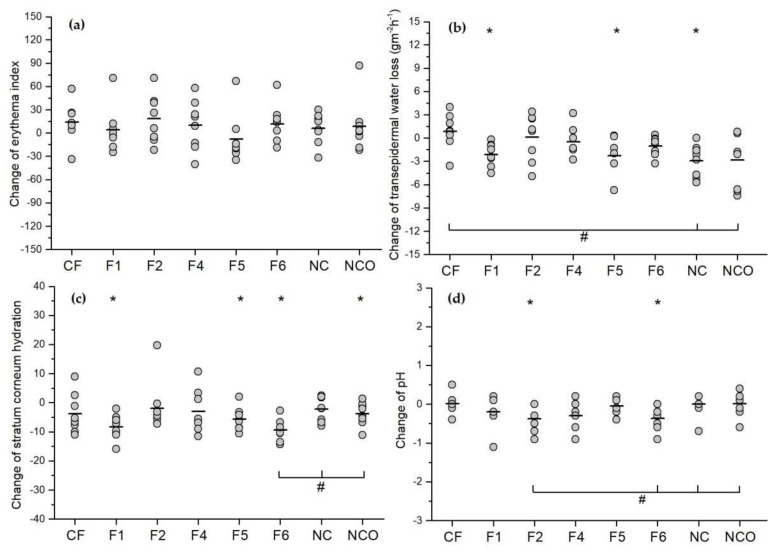
Influence of the developed and control film-forming formulations on (**a**) EI, (**b**) TEWL, (**c**) SCH and (**d**) pH value. Parameters are expressed as an absolute change in values obtained 1 h after occlusion removal vs. baseline values for the investigated samples, non-treated control under occlusion (NCO) and without occlusion (NC). * *p* < 0.05 compared to baseline values (Student’s *t*-test or Wilcoxon Signed-Rank Test, depending on the nature of the data); # *p* < 0.05 compared to both controls (ANOVA followed by Tukey post hoc test or Kruskal-Wallis test followed by Mann-Whitney U test, depending on the nature of the data).

One hour after occlusion removal, no adverse reactions were observed during visual inspection of the skin treated with all tested FFS. Additionally, no significant change was observed in EI values compared to baselines and non-treated controls (Figure 2a), indicating acceptable skin tolerability for all the investigated samples.

Contrary to the EI parameter, the type of polymer used for the preparation of the investigated samples significantly affected the TEWL values during the study. Samples F1 and F5, prepared using Eudragit^®^ RS PO and Eudragit^®^ NE 30 D, respectively, led to a significant decrease in this parameter related to baseline values (*t*-test, *p* < 0.05). Precisely, the TEWL decrease was between 40 and 50%, suggesting that the formed films partially blocked the evaporation of surface water, exerting a semi-occlusive effect on the skin [36]. However, it should be noted that all the tested samples led to a decrease in SCH compared to baselines whereas a pronounced decrease was detected in the case of F1, F5 and, particularly, F6 sample (*t*-test or Wilcoxon test, depending on the nature of the data, *p* < 0.05). At a first glance, this finding was not entirely in agreement with the results of TEWL measurements as a certain increase in SCH was to be expected. However, one should bear in mind that the semi-occlusive films are not non-permeable and allow more water transport compared to the occlusive ones [36]. Hence, the decrease in skin hydration could be attributed to a relatively high amount of volatile solvents (ethanol/isopropyl alcohol) present in the developed FFS. Likewise, it appears that the addition of plasticizers such as TEC and MCT in corresponding formulations (F2 and F4), resulting in films of higher thickness, somewhat alleviated the dehydration effect of the used solvents (no significant changes in SCH values were observed, Wilcoxon test, *p* > 0.05). Additionally, when comparing the TEWL and SCH results obtained for investigated formulations with both non-treated controls (NC and NCO), no statistically significant differences (ANOVA or Kruskal-Wallis test, *p* > 0.05) were observed (with the exception of F6; Figure 2b,c). However, it should be noted that the values of the measured biophysical parameters slightly decreased on both non-treated sites, while the occlusion itself exerted a marginal effect on the skin during the study. Nevertheless, this was not surprising because many factors, either environment- or individual-related, are reported to influence TEWL and SCH measurements in such a way [3].

On the other hand, interestingly, the CF sample with PVA as the film-forming polymer led to a significant increase in TEWL compared to both non-treated controls (ANOVA, *p* < 0.05). As no significant changes in the SCH levels were detected during the study, the observed TEWL increase could be attributed to the hygroscopic properties of PVA rather than the alterations in skin barrier integrity. Finally, when analyzing the pH values (Figure 2d), it was notable that only F2 and F6 samples led to a statistically significant change in this parameter related to their baselines (*t*-test, *p* < 0,05) and to both non-treated controls (ANOVA, *p* < 0,05). However, it should be emphasized that these two formulations induced, although statistically significant, a slight pH drop after 24 h occlusion (from 5.3 ± 0.3 to 4.9 ± 0.4 for both samples). Therefore, it is reasonable to assume that the observed small differences in pH values (within 0.5 pH units) would not significantly affect the in vivo performance of the prospective drugs. Interestingly, the F4 formulation, despite the slightly higher pH value, did not exert a statistically significant effect on the skin pH value. Overall, all the findings of the first phase of the in vivo study provide additional proof of the mild nature of the tested FFS.

**Figure 3:**
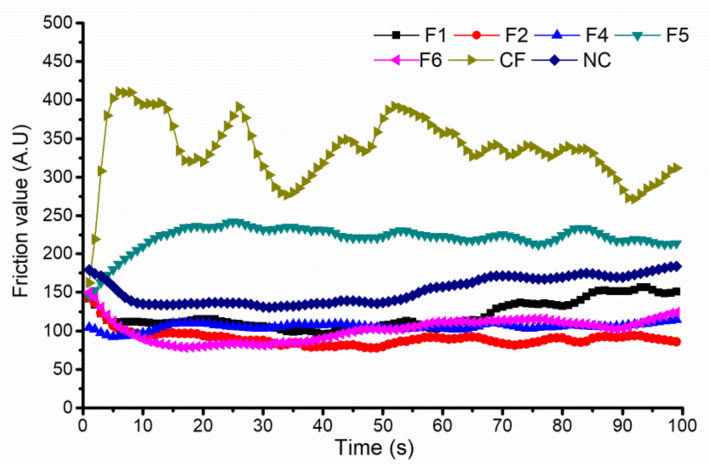
Friction curves obtained 5 min after application of the developed film-forming systems on the volar forearm area in comparison with the bare skin (non-treated control). Data represent the mean value obtained for 8 volunteers.

The distinct transformation of the FFS upon application along with their potential to reduce drug dosing frequency imply the need for a supplementary in vivo assessment. In order to gain further insight into the interactions between the skin and the generated films, a tribological study was subsequently performed. Generally, the resistance to friction (substantivity) of film-forming systems is of paramount importance regarding the prospective therapeutic effect of incorporated drugs, determining the residence time, release kinetics and skin absorption efficiency [37]. The friction curves obtained 5 min after application of the tested FFS on the volunteers’ volar forearms in comparison with the non-treated control are depicted in Figure 3. Firstly, it can be seen that during the friction procedure (100 s), formulations F1, F2, F4 and F6 exhibited certain lubricant behavior on the skin, with friction values lower than for the non-treated control. However, the time needed to reach the plateau and its value and length were highly dependent on the polymer type and its concentration (Figure 3). In the case of F1 formulation, prepared with hydrophobic polymer RS (8.5%), the plateau was reached after approximately 5 s and lasted until approximately 20 s. After 40 s, a slight gradual increase in the friction coefficient was observed. This surface phenomenon can be explained by solvent evaporation during the measurement and some degree of stickiness of the residual film [38]. Interestingly, the inclusion of an additional polymer such as HPC (sample F6) prolonged the time required to reach the plateau (approximately 20 s) and its length (up to approximately 40 s), simultaneously causing the lower values of the friction coefficient. This could be attributed to a significantly higher film thickness of the F6 formulation compared to F1 and an improved lubricating effect. Likewise, it should not be forgotten that smoother films require less force from the probe, resulting in a lower friction value. However, this assumption needs further confirmation, preferably by analyzing the surface roughness with appropriate techniques such as AFM. In the case of samples F2 (prepared with 10% of polymer RS and 2% of TEC as the plasticizer) and F4 (3.5% of HPC and 0.7% of MCT), the lubricant effect allowed the maintenance of a relatively stable and low friction coefficient, showing the curves with little evolution during friction procedure. It is interesting to note that, although these two formulations exhibited different drying times at 32 °C (4.6 ± 0.1 min for F2 and 13.5 ± 0.9 min for F4), no significant difference regarding the friction coefficient was observed. On the other hand, quite unexpectedly, the application of F5 (prepared with 6% of the polymer NE) and the control formulation (prepared with 10% of PVA) was accompanied by a significantly higher friction value compared to the non-treated control over time. Commonly, the friction value is higher at the beginning of measurements and gradually declines to the plateau value [39]. As no correlation was detected with the drying time of the films at 32 °C and other physico-mechanical properties of the films evaluated, the erratic curves (particularly for CF sample) could be attributed to the increased adhesion force between the contact material (Frictiometer^®^ Teflon probe) and the films formed after dynamic metamorphosis process. Analysis of these tribological curves allows us to presume the importance of the moment the plateau is reached as the onset of the so-called secondary formulation within FFS metamorphosis. The tertiary state is still best described by the completion of the film’s drying time.

**Figure 4:**
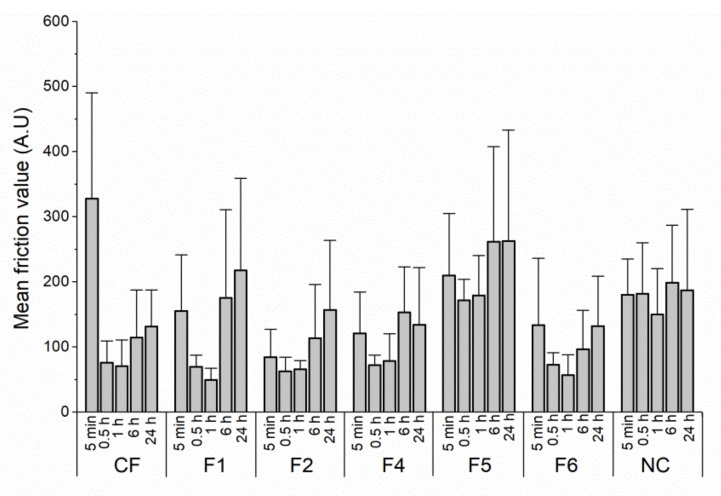
The changes in mean friction value on skin areas treated with developed film-forming systems and non-treated control over 24 h.

Due to the lack of studies dealing with skin friction analysis after the application of FFS, it was also interesting to evaluate the long-term tribological behavior after applying the selected samples onto the skin. The changes in mean friction values on areas treated with the tested film-forming systems and non-treated control over 24 h are shown in Figure 4. Over 1 h, the mean friction value gradually declined for all the tested formulations due to the solvent evaporation and the formation of homogeneous, fine-structured films at the skin surface (the mean friction coefficient was significantly lower relative to the non-treated control, except for the sample F5). Additionally, it should be noted that the most pronounced decrease in the friction coefficient was detected for the sample CF. This finding is quite expected, considering the study reported by [40]. Precisely, the authors observed a significant increase in the skin friction coefficient 5 min after application of distilled water due to the presence of a capillary adhesion force between the probe and the skin, which reversed back to the initial state after around 30 min. Hence, because the sample CF contained the highest quantity of water (89.5%), it appears that the presence of water increased the frictional resistance between the probe and the skin instead of lubricating the contact. The average friction coefficient for CF significantly declined after 1 h due to the complete water evaporation from the corresponding film at skin surface.

After 6 h, the mean friction value gradually increased for all the tested formulations, clearly implying the changes in the films’ structure and their substantivity. In other words, it appears that the value of the skin friction parameter slowly reached the value of the non-treated control. Likewise, during the visual inspection of the skin surface, it was observed that in the case of samples F4 and CF, based solely on hydrophilic film-formers, the films gradually detached from the skin and were not able to persevere on the skin surface over 24 hours. Our findings are in accordance with those of Monica et al. [37] who showed that polymethacrylate samples required a higher force to detach from the skin compared to HPC-based samples and, consequently, would last longer in body areas with higher friction. However, it is important to emphasize that no statistically significant difference was observed between the samples F2 (persistent and consistent film) and F4 (inconsistent film, detached from the skin) regarding mean friction value determined 24 h after sample application (156.5 ± 107.1 and 133.9 ± 87.8, respectively). The sample F5 based on the hydrophobic neutral polymer NE showed somewhat different tribological behavior to other samples, maintaining relatively high friction throughout the experiment (Figure 3 and Figure 4). This could be partly attributed to the relatively lower bioadhesive properties of the NE polymer, and partly to the fact that the generated film significantly elevated SCH as the skin biophysical parameter directly responsible for the changes in physical and mechanical properties of the stratum corneum [41], subsequently recorded by the sensitive Frictiometer probe.

**Figure 5:**
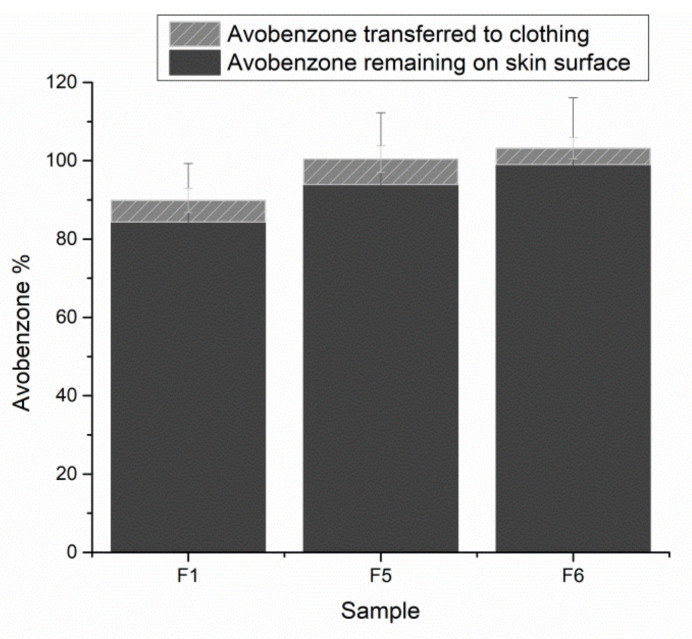
In vivo substantivity test depicting the ratio of the model active substance fixed on the skin within the generated film vs. the one transferred to the volunteers’ clothing.

These findings implied the need for the inclusion of another technique able to study film substantivity more profoundly. For that purpose, three samples were selected: F1 based on the hydrophobic cationic polymer RS; F5 based on hydrophobic but neutral polymer NE; and sample F6 combining polymer RS with hydrophilic HPC. This final part of the in vivo study proved high skin substantivity of each FFS tested (Figure 5), allowing only 4.13–6.50% of the formulation to be transferred to the simulated clothing. Despite the fact that substantivity is commonly presumed for the cationic materials, sample F5 performed better than sample F1 in this study. However, the highest skin substantivity was attributed to the sample F6 (99.07 ± 16.99%), confirming the optimal 4:1 ratio of the hydrophobic RS and hydrophilic HPC film-formers indicated rather early during the screening of FFS physico-mechanical properties. Relatively high standard deviations appear to be characteristic of these studies, reflecting the variations in physical activity of the volunteers [29].

## 4. Conclusions

Due to the rapid solvent displacement upon skin application, FFS go through a substantial transformation, morphing through several states. As prospective (trans)dermal delivery systems, a skin performance study of FFS must go beyond usual characterization techniques to include certain aspects of tribology and substantivity. This paper used several known film-forming polymers of diverse properties to test the hypothesis that a selection of physico-mechanical characterization techniques followed by targeted in vivo assays may discern optimal FFS for pharmaceutical use and complement their quality target product profiles. The study has shown that such systems are generally non-irritant, non- to semi-occlusive and do not disturb the natural mechanical barrier of the skin while possessing the substantivity required for sustaining the active ingredient at the target site for a sufficient amount of time, ultimately reducing the application frequency. Finally, the inclusion of the tribological study appears to be of special interest for FFS, revealing more fundamental interactions at play on the skin surface, and even being able to discern the primary stage from the secondary stage of the system’s metamorphosis.

## Figures and Tables

**Table 1:** Percentage composition of the constituents within the investigated film-forming systems.

Function	Constituents	(%, *w/w*)
Polymer or combination of polymers	Eudragit^®^ RS	8.5–19.0
	Eudragit^®^ NE 30 D	5.0–10.0
	Klucel^®^ GF	2.5–5.0
	Eudragit^®^ RS/Klucel^®^ GF	4/1
Plasticizer/penetration enhancer	TEC ^1^	20 *
	TBC ^2^	20 *
	Propylene glycol	20 *
	Glycerol	20 *
	MCT ^3^	20 *
	Polysorbate 80	0.3–3
Solvent or mixture of solvents	Propylene glycol/EtOH ^4^/water ^5^	1–5/73.4–92.4/1.3–12.7
	EtOH/water	73.4–86.0/3.3–8.2
	Isopropyl alcohol/water	73.0–87.5/3.3–12.7
	EtOH/water/ethyl acetate	74.0/10.6/1

* %, *w/w* of the dry polymer. ^1^ TEC = triethyl citrate; ^2^ TBC = tributyl citrate; ^3^ MCT = medium-chain triglycerides; ^4^ EtOH = ethanol 96%, ^5^ water = purified water.

**Table 2:** Composition of the selected in situ film-forming formulations.

Excipients	Composition (%, *w/w*)					
	F1	F2	F3	F4	F5	F6
Eudragit^®^ RS PO	8.5	10.0	17.5	−	−	4.0
Eudragit^®^ NE 30 D	−	−	−	−	6.0	−
Klucel^®^ GF	−	−	−	3.5	−	1.0
TEC ^1^	−	2.0	−	−	−	−
MCT ^2^	−	−	−	0.7	−	−
Propylene glycol	1.0	−	3.5	−	−	0.5
EtOH ^3^	86.7	84.7	73.4	95.8	−	92.9
Isopropyl alcohol	−	−	−	−	85.0	−
Polysorbate 80	1.0	−	−	−	−	0.3
Water ^4^ up to	100	100	100	−	100	100

^1^ TEC = triethyl citrate; ^2^ MCT = medium-chain triglycerides; ^3^ EtOH = ethanol 96%, ^4^ water = purified water.

**Table 3:** Evaluation of the selected in situ film-forming formulations (mean ± SD, *n* = 3).

Formulation	Drying Time of the Film (32.0 ± 0.1 °C) (min)	Drying Time of the Film (25 ± 2 °C) (min)	Film Surface (mm^2^)
F1	6.6 ± 0.4 ^a^	24.0 ± 0.6 ^e^	223.0 ± 21.2 ^d^
F2	4.6 ± 0.1 ^b^	32.0 ± 2.5 ^f^	121.0 ± 2.0 ^d^
F3	5.3 ± 0.2 ^c^	22.0 ± 2.0 ^e^	79.0 ± 1.5 ^g^
F4	13.5 ± 0.9 ^d^	40.0 ± 0.6 ^d^	24.0 ± 1.2 ^d^
F5	6.3 ± 0.6 ^a^	48.0 ± 1.5 ^d^	72.0 ± 5.2 ^g^
F6	4.6 ± 0.4 ^b^	32.0 ± 2.0 ^f^	83.0 ± 1.5 ^g^

^a^ *p* < 0.05 compared to F2, F4 and F6 formulations. ^b^ *p* < 0.05 compared to F1, F4 and F5 formulations. ^c^ *p* < 0.05 compared to F4. ^d^ *p* < 0.05 compared to all tested formulations. ^e^ *p* < 0.05 compared to F2, F4, F5 and F6 formulations. ^f^ *p* < 0.05 compared to F1, F3, F4 and F5 formulations. ^g^ *p* < 0.05 compared to F1, F2 and F4 formulations.

**Table 4:** Folding endurance value, film thickness and pH of the corresponding film-forming systems (mean ± SD, *n* = 3).

Formulation	Folding Endurance Value	Film Thickness (mm)	pH Value
F1	112.0 ± 2.9 ^a^	0.007 ± 0.002 ^f^	6.9 ± 0.1 ^g^
F2	54.0 ± 3.2 ^b^	0.021 ± 0.001 ^d^	6.1 ± 0.1 ^d^
F3	95.0 ± 1.5 ^c^	0.096 ± 0.002 ^d^	6.7 ± 0.1 ^e^
F4	78.0 ± 1.5 ^c^	0.046 ± 0.001 ^d^	8.3 ± 0.1 ^d^
F5	56.0 ± 3.5 ^b^	0.011 ± 0.002 ^d^	7.5 ± 0.1 ^d^
F6	105.0 ± 9.0 ^e^	0.033 ± 0.003 ^f^	6.6 ± 0.1 ^c^

^a^ *p* < 0.05 compared to F2, F3, F4 and F5. ^b^ *p* < 0.05 compared to F1, F3, F4 and F6. ^c^ *p* < 0.05 compared to F1, F2, F4 and F5. ^d^ *p* < 0.05 compared to all tested formulations. ^e^ *p* < 0.05 compared to F2, F4 and F5. ^f^ *p* < 0.05 compared to F2, F3, F4 and F6. ^g^ *p* < 0.05 compared to F2, F4, F5 and F6.

## Supplementary Materials

The following supporting information can be downloaded at: https://www.mdpi.com/article/10.3390/pharmaceutics14020223/s1, Supplementary material on the preformulation study: Table S1. Composition (% *w/w*) of the FFS that passed the first tier of sample number refinement; Table S2. Composition (%, *w/w*) of the FFS that passed the second tier of refinement.
