# Supplementary material for: Simultaneous Physico-Mechanical and In Vivo Assessment towards Factual Skin Performance Profile of Topical Polymeric Film-Forming Systems
Source: Pharmaceutics. 2022 Jan 18;14(2):223. doi: 10.3390/pharmaceutics14020223 (PMC8877452; doi:10.3390/pharmaceutics14020223)
Supplement: Supplementary file 1 [file pharmaceutics-14-00223-s001.zip › pharmaceutics-1536733-supplementary.pdf]

pharmaceutics

Supplementary Material: Simultaneous Physico-Mechanical and In Vivo Assessment Towards Factual Skin Performance Profile of Topical Polymeric Film-Forming Systems

Mirjana D. Timotijević, Tanja Ilić, Snežana Savić and Ivana Pantelić *

Preformulation study leading to refinement of the number of film-forming systems entering the in vivo studies with human volunteers

A pre-formulation study based on the comprehensive literature search resulted in preparation of film-forming systems (FFS) in the form of polymer solutions or dispersions (total of 55 formulations), with the following types of polymers: Eudragit® RS PO, Eudragit® NE 30 D, Klucel® GF, or their combinations.

The effect of adding a variety of plasticizers such as triethyl citrate (TEC), tributyl citrate (TBC), propylene glycol or glycerol, with optional use of a penetration enhancer such as medium chain triglycerides Miglyol 812® (MCT) or a nonionic surfactant (Polysorbate 80) was studied. Additionally, the most suitable type of solvent or solvents systems in FFS was evaluated. The mentioned excipients were assayed in the concentrations specified below.

Polymers which form thin, in situ polymeric films:

1. Klucel®GF (hydroxypropyl cellulose, Caesar & Loretz GmbH, Hilden, Germany) at concentrations of 2.5%, 3.5%, 4.0% and 5.0%;
2. Eudragit® NE 30 D (poly (ethyl acrylate-co-methyl methacrylate) 2:1 (polyacrylate dispersion 30% Ph. Eur.), Evonik Rohm GmbH, Darmstadt, Germany) at concentrations of 5.0%, 5.5%, 6.0%, 6.5%, 7.5%, 8.5% and 10.0%;
3. Eudragit® RS PO (poly (ethyl acrylate-co-methyl methacrylate-co-trimethylammonioethyl methacrylate chloride), i.e. copolymer type B, Ph. Eur., Evonik Rohm GmbH, Darmstadt, Germany) at concentrations of 8.5%, 9.0%, 10.0%, 12.0%, 12.5%, 15.0%, 17.5%, 18.0% and 19.0%; and
4. Combinations of polymer Eudragit® RS PO with Klucel® GF at concentrations of 1% and 4% each.

Plasticizers: triethyl citrate, tributyl citrate, propylene glycol or glycerol were added in concentration of 20% (*w*/*w*) relative to the weight of the polymer.

Penetration enhancers**:** lipidic – such as medium chain triglycerides Miglyol 812®, or hydrophilic – such as propylene glycol, were added in concentration of 20% (*w*/*w*) relative to the weight of the used polymer, while the addition of an amphiphilic penetration enhancer was optionally assessed – non-ionic surfactant Polysorbate 80 in concentrations of 0.3%, 0.5%, 1.0% and 3.0 (*w*/*w*).

Solvent or solvent system: ethanol (96%, *v*/*v*) or ethanol/water, propylene glycol/ethanol/water, isopropyl alcohol/water, ethanol/water/ethyl acetate were varied.

The prepared 55 formulations were submitted through the following characterization methods:

1. Film drying time at 23 ± 2 °C and at 32 ± 0.1 °C
2. Film thickness
3. Sensory properties (namely appearance and stickiness)
4. pH of FFS
5. Flexibility/mechanical resistance (i.e. folding endurance)

(*for the detailed description of the applied methods, the reader is referred to the Methods section of the manuscript)

Samples containing Klucel® GF: The concentration of the polymer Klucel® GF was within the range from 2.5 to 5.0%. It was found that while increasing the concentration of

this hydrophilic polymer from 2.5% to 3.5% a significant increase of the film drying time at 32 °C, as well as at the room temperature occurred. After a 3-month storing period at room temperature, consistency of the formulations with 4% and 5% of Klucel® GF was markedly increased, transitioning to potential film-forming gel formulation which is a form beyond the scope of this research. The sample transformation from film-forming solution to film-forming gel was considered to be one of the criteria for exclusion from further evaluation.

Samples containing Eudragit® NE: Increasing the concentration of the polymer Eudragit® NE from 5.0% to 8.5% prolonged the drying time of the film both at the room (23 °C) and skin temperature (32 °C).

Samples containing Eudragit® RS PO: Increasing the concentration of the polymer Eudragit® RS PO from 10% to 17.5% did not significantly affect the drying time of the film at 32 °C, while the presence of Polysorbate 80 at a concentration of 1% prolonged the film drying time at 32 °C to 6 minutes, 40 seconds.

It can be concluded that the type and concentration of the chosen polymer has a determinant effect on film drying time, but it was also discovered that the presence of Polysorbate 80 has an influence on film drying time despite the polymer concentration.

First tier of sample refinement:

After considering the following exclusion criteria, the first tier of sample number reduction was performed:

1. drying time at room temperature > 60 min
2. drying time at skin temperature > 15 min
3. high or medium stickiness of the formed films at 32 °C and at the room temperature
4. low folding endurance value (< 5 folding times lead to visible changes or cracks on the formed film surfaces)
5. film thickness > 0.1 mm
6. pH < 5.0 or > 8.5

After applying these exclusion criteria, the initial 55 formulations were reduced to 20 samples, outlined in the Supplementary Table 1

Table S1. Composition (% *w/w*) of the FFS that passed the first tier of sample number refinement.

Formulation	Type of polymer	Conc. of polymer (%)	Plasticizer	Conc. of plasticizer (%)	Solvent	Conc.of solvent (%)	Polysorbate 80 (%)
F1	Eudragit® RS	8.5	Propylene glycol	1.0	Ethanol/Water	86.7/2.8	1.0
F2	Eudragit® RS	10.0	TEC	2.0	Ethanol/Water	84.7/3.3	/
F3	Eudragit® RS	17.5	Propylene glycol	3.5	Ethanol/Water	73.4/5.6	/
F4	Klucel® LF	3.5	MCT	0.7	Ethanol	95.8	/
F5	Eudragit® NE 30 D	6.0	/	/	Isopropyl alcohol/Water	85.0/9.0	/
F6	Eudragit® RS/Klucel ®GF	4.0/1.0	Propylene glycol	0.5	Ethanol/Water	92.9/1.3	0.3
F7	Klucel® GF	2.5	MCT	0.7	Ethanol	96.8	/
F8	Klucel® GF	2.5	Propylene glycol	0.7	Ethanol	96.8	/
F9	Eudragit® RS	12.5	TEC	2.5	Ethanol/Propylene glycol/ Water	75.9/5.0/4.1	/

F10	Eudragit® RS	12.0	MCT	2.4	Ethanol/Water	81.6/4	/
F11	Eudragit® RS	12.5	TEC	2.5	Ethanol/Water	80.8/4.2	/
F12	Eudragit® RS	12.5	Propylene glycol	2.5	Ethanol/Water	82.0/2.5	0.5
F13	Eudragit® NE 30 D	8.5	/	/	Ethanol/Water	78.8/12.7	/
F14	Klucel® GF	3.5	Propylene glycol	1.0	Ethanol	92.5	3.0
F15	Eudragit® RS	8.5	Propylene glycol	1.0	Ethanol/Water	74.8/12.7	3.0
F16	Eudragit® RS	15	MCT	3.0	Ethanol/Water	77.0/5.0	/
F17	Eudragit® RS/ Klucel®GF	4.0/1.0	/	/	Ethanol/Propylene glycol/ Water	88.7/5.0/1.3	/
F18	Eudragit® RS	8.5	Propylene glycol	1.0	Ethanol/Water	87.4/2.8	0.3
F19	Eudragit® RS	17.5	MCT	3.5	Ethanol/Water	75.0/4.0	/
F20	Eudragit® NE	5.5	/	/	Ethanol/Water	86.3/8.2	/

Second tier of sample refinement:

Subsequently, after considering more stringent exclusion criteria, the second tier of sample number reduction was performed:

low folding endurance value (< 15 folding times lead to visible changes or cracks on the formed film surfaces)

After applying the additional exclusion criteria, the selection of 6 formulations for *in vivo* studies was performed, detailed in Supplementary Table 2 (also Table 2 of the manuscript).

Table S2. Composition (%, *w/w*) of the FFS that passed the second tier of refinement.

**Excipients**	**Composition (%, *w/w*)**					
	**F1**	**F2**	**F3**	**F4**	**F5**	**F6**
Eudragit® RS PO	8.5	10.0	17.5	-	-	4.0
Eudragit® NE 30 D	-	-	-	-	6.0	-
Klucel® GF	-	-	-	3.5	-	1.0
TEC	-	2.0	-	-	-	-
MCT	-	-	-	0.7	-	-
Propylene glycol	1.0	-	3.5	-	-	0.5
Ethanol, 96% V/V	86.7	84.7	73.4	95.8	-	92.9
Isopropyl alcohol	-	-	-	-	85.0	-
Polysorbate 80	1.0	-	-	-	-	0.3
Purified water up to	100	100	100	-	100	100

From all the polymers included in this study (Klucel® GF, Eudragit® RS PO, Eudragit® NE 30 D), also including the polymer combination Eudragit® RS PO with Klucel® GF, the best results of placebo FFS systems in terms of observed physico-chemical and mechanical parameters were obtained by the polymer Eudragit® RS PO, at the concentrations of 8.5%, 10% and 17.5% (*w/w*), and the polymer combination of Eudragit® RS PO with Klucel® GF (4%/1%).

This preformulation study revealed that the presence of the non-ionic surfactant Polysorbate 80 at a concentration of 1% improves the flexibility and the mechanical strength of the film, even at a lower concentration of the polymer Eudragit® RS PO (8.5%) and in the presence of propylene glycol (1%). Increasing the concentration of the polymer Eudragit® RS PO from 10% to 17.5% increases the value of the mechanical resistance of the

film, but the highest value for the film mechanical resistance was obtained by a formulation containing Polysorbate 80 at a concentration of 1%, and a lower concentration (8.5%) of the polymer Eudragit® RS PO. The type and concentration of plasticizer has a strong influence on film mechanical properties and film stickiness.

Within the formulations, the most suitable plasticizers for the dispersions with Eudragit® RS were shown to be propylene glycol or TEC (20% relative to the dry weight of polymer). The presence of glycerol as a plasticizer did not provide sufficient flexibility and mechanical resistance of the *in situ* formed films. The same applies to the presence of MCT. Regarding the most suitable solvent for the polymers Eudragit® RS and Klucel® GF, this appeared to be ethanol (with addition of water in the case of Eudragit® RS). The use of isopropyl alcohol as a solvent in combination with the polymer Eudragit® NE (6%, 8.5%) afforded a low stickiness of the films at 32 °C and at a room temperature, but the flexibility of the formed film was also reduced. Considering the drying times and film flexibility and mechanical resistance, optimal concentration of the polymer Eudragit® NE was shown to be 6.0% in the solvent system isopropyl alcohol/water.

The thickness of the formed films was in the range of 0.007 to 0.096 mm. The highest value of the film thickness was shown by formulation F3 with 17.5 % of the polymer Eudragit® RS PO, while the lowest value for film thickness is obtained by formulation F1 with the polymer Eudragit® RS PO at the concentration of 8.5 % (0.007 mm). The formulation with the polymer Eudragit® RS at 17.5 % had a higher value of film thickness (0.096 mm) compared to the formulation with 10 % Eudragit RS (0.021 mm). The formulation with the polymer Eudragit® RS at the concentration of 15 % had a film thickness of 0.084 mm. Increasing the concentration of the polymer Eudragit® NE from 5.0 % to 8.5 % had a slight decreasing effect on the film thickness (from 0,014 mm to 0,010 mm). The formulation with the polymer Klucel® GF at the concentration of 3.5% provided a higher film thickness (0.046 mm) compared to the formulation with 2.5% Klucel® GF (0.032 mm).

The results show that thickness of the *in situ* formed films in the FFS is directly dependent and in correlation with the chosen type of polymer and its concentration in the case of Eudragit® RS and Klucel® GF, indicating that with an increase of the concentration of said polymers there is also a significant increase of the film thickness. In the case of Eudragit® NE, the increase of the concentration of polymer leads to a slight decrease of the film thickness. The results have shown that the pH values of film-forming solutions/dispersions were in the range from 5.5-8.5. More specifically, pH values of FFS with polymer Eudragit® NE were in the range from 7.6-7.9.

The obtained results were sufficient to imply skin safety of the developed FFS, and obtain suitable ethical approval.
